# Safety profiles of current and emerging neoadjuvant immune-modulatory and combination strategies in borderline resectable stage III non-small cell lung cancer: a narrative review

**DOI:** 10.3389/fphar.2026.1929734

**Published:** 2026-10-23

**Authors:** Nabih Berjaoui, Yee Tung (Ruby) Siu, Andrea Bille, Akshay J. Patel

**Affiliations:** 1 Department of Thoracic Surgery, Guy’s and St Thomas’ NHS Foundation Trust, London, United Kingdom; 2 Department of Medicine, London Northwest University Healthcare NHS Trust, London, United Kingdom; 3 Institute of Immunology and Immunotherapy, University of Birmingham, Birmingham, United Kingdom

**Keywords:** borderline patients, checkpoin control pathway, lung cancer, surgery, toxicity

## Abstract

Borderline resectable stage III non-small cell lung cancer (NSCLC) has no consensus definition. In an international multidisciplinary survey of stage III TNM subsets, no agreement on resectability was reached for 11 of 37 subsets, and the multisocietal consensus exercise conducted alongside it left the same anatomical configurations unresolved. This review adopts those non-consensus subsets as its working definition and asks how the safety profiles of current and emerging neoadjuvant immune-modulatory and combination strategies, spanning PD-1 and PD-L1 blockade, emerging checkpoint and immune-metabolic targets, antibody-drug conjugates and bispecific antibodies, bear on surgical decision-making within them. Six phase 3 trials now support neoadjuvant or perioperative PD-1 or PD-L1 blockade with platinum doublet chemotherapy in resectable disease, with pathological complete response rates of 17.2%–40.7% and event-free survival hazard ratios of 0.40–0.68. Grade 3 or higher treatment-related adverse events ranged from 32.5% to 73.0%, immune-mediated adverse events from 25.3% to 42.1% where an aggregate was reported, and treatment-related cancellation of surgery remained below 2% in every trial reporting it. Most patients who never reached resection did so for reasons other than toxicity. Among emerging targets, monospecific TIGIT blockade has failed in three randomised settings and the most advanced TIM-3 programme was discontinued on company-reported evidence, which recontextualises the earlier phase 2 signals for both classes. NKG2A, CD73 and TROP2-directed combinations have produced neoadjuvant pathological response signals without loss of surgical feasibility, although each rests on a single-arm platform cohort of fewer than 80 patients. One PD-1 and VEGF bispecific antibody has positive peer-reviewed phase 3 data in advanced disease and no neoadjuvant data. The operative question in borderline resectable disease is not whether response rates rise but whether toxicity, surgical delay and functional decline shift the risk-benefit balance in patients already marginal for resection. Answering it requires prospective cohorts in which borderline status is defined before treatment, of which MDT-BRIDGE and NEOINSPIRE are the first.

## 1 Introduction

Non-small cell lung cancer (NSCLC) accounts for approximately 85% of lung cancers, and lung cancer remains the leading cause of cancer death worldwide (Sung et al., 2021). Stage III disease makes up roughly a quarter of new diagnoses and is the most anatomically heterogeneous of the stage groupings, spanning a small peripheral tumour with one involved mediastinal station through to bulky multistation nodal disease with mediastinal infiltration (Goldstraw et al., 2016; Detterbeck et al., 2017). That heterogeneity is not a classification detail. It determines whether an operation is offered at presentation, whether it is offered after induction therapy, or whether radiotherapy carries the curative intent.

Treatment allocation in stage III still rests on a binary judgement of resectable or unresectable. At one end of the spectrum, definitive concurrent chemoradiation followed by consolidation durvalumab is standard for unresectable disease (Antonia et al., 2017). At the other, ASCO guidance sets out the conditions under which resection should form part of multimodality treatment (Daly et al., 2022). Between them sits a population about which multidisciplinary teams disagree with each other. The EORTC Lung Cancer Group has approached this twice: a multisocietal Delphi consensus that reviewed 105 real-world stage III cases and produced a technical resectability definition for trial inclusion criteria (Dingemans et al., 2026), and an international multidisciplinary survey in which no consensus was reached for 11 of 37 stage III TNM subsets (Houda et al., 2025). Disagreement clustered around small primary tumours with multistation N2, larger primary tumours with single-station N2, and invasive T4 disease with limited nodal involvement (Houda et al., 2025). Technical resectability is in any case only one axis. Functional operability, meaning pulmonary reserve, cardiopulmonary fitness and frailty, is assessed separately and constrains what an anatomically resectable tumour permits (Brunelli et al., 2009).

Systemic therapy has moved in front of the operation. In a meta-analysis of randomised trials in resectable NSCLC, the timing of checkpoint inhibition was the dominant explanatory variable for overall survival benefit, with neoadjuvant and perioperative administration outperforming adjuvant-only administration (Patel et al., 2025). Six phase 3 trials now support preoperative PD-1 or PD-L1 blockade combined with platinum doublet chemotherapy in resectable stage II to III disease (Forde et al., 2022; Wakelee et al., 2023; Heymach et al., 2023; Cascone et al., 2024; Lu et al., 2024; Yue et al., 2025a). Each reported higher pathological complete response (pCR) and longer event-free survival (EFS) than chemotherapy alone. None was designed around the population in which the operative decision is contested, and none reported outcomes for a prospectively defined borderline subgroup.

The surgical literature has so far read these trials as reassurance: resection rates held, R0 rates held, and treatment-related cancellation of surgery was rare. Those denominators consist of patients whom investigators had already judged fit for and suitable for resection at enrolment. This review examines the safety profiles of current and emerging neoadjuvant immune-modulatory and combination strategies through the lens of surgical decision-making in borderline resectable stage III disease, and appraises which emerging targets carry evidence sufficient to justify preoperative exposure in a patient who could be operated on today. The scope deliberately extends beyond checkpoint inhibitors in the strict pharmacological sense to antibody-drug conjugates and VEGF-directed bispecific antibodies, because these agents are entering the same preoperative platform trials, are combined with or positioned against the same PD-1 based chemoimmunotherapy comparators, and raise the same surgical questions of toxicity, delay and functional decline that this review exists to answer. Throughout, borderline resectable means anatomically resectable stage III subsets for which multidisciplinary consensus on upfront surgery was not reached (Dingemans et al., 2026; Houda et al., 2025).

## 2 Historical framework and the evolving definition of resectability

Surgery for stage III NSCLC lost ground largely on the strength of one trial. In INT0139, induction chemoradiation followed by resection did not improve overall survival over definitive chemoradiation, although 5-year survival was 27.2% in the surgical arm against 20.3% without surgery (Albain et al., 2009). Excess perioperative mortality concentrated in patients undergoing pneumonectomy. Two decades of practice followed in which N2 involvement was treated in many units as a contraindication to primary resection, and the stage III evidence base became predominantly a radiotherapy evidence base (Detterbeck et al., 2017; Daly et al., 2022). That trial predates the current staging framework, contemporary lung-sparing anatomical resection, and any systemic regimen with a pathological response rate above single figures.

The anatomical vocabulary has since become finer. The ninth edition TNM proposals subdivide N2 into N2a, single-station involvement, and N2b, multistation involvement, on the basis of divergent survival (Rami-Porta et al., 2024). The distinction carries surgical weight, because single-station N2 with a resectable primary is a different operation, and a different risk of incomplete resection, from multistation N2 with mediastinal infiltration. The eighth edition remains the staging reference for the trials discussed here (Goldstraw et al., 2016). Within T4, invasion of the carina, a vertebral body or a great vessel is not the same problem as a tumour classified T4 by size or by an additional nodule in a different ipsilateral lobe, yet both enter trials under one descriptor (Goldstraw et al., 2016; Rami-Porta et al., 2024).

The working definition used here is drawn from the EORTC exercises rather than from any trial protocol. The multisocietal Delphi consensus reviewed 105 real-world stage III cases across three rounds and classified most TNM subsets as technically resectable or not, reserving the unresectable label largely for invasive T4 disease with multistation or bulky nodal involvement and for any N3 disease (Dingemans et al., 2026). The accompanying multidisciplinary survey found that agreement failed for 11 of 37 subsets (Houda et al., 2025). Those non-consensus subsets, set out in Table 1, are what this review means by borderline resectable. The label is anatomical and procedural by design: it describes a tumour that a competent multidisciplinary team might or might not take to theatre, not a tumour of a particular size or a patient of a particular fitness.

**TABLE 1:** Anatomical subsets in which multidisciplinary consensus on resectability was not reached in the EORTC Lung Cancer Group consensus and survey, and their representation in the six pivotal phase 3 neoadjuvant or perioperative trials. Evidence strength refers to whether the subset was analysed directly, inferred from a broader stage III population, or not reported.

Anatomical subset	EORTC classification (Dingemans et al., 2026, Houda et al., 2025)	Representation in pivotal phase 3 trials	Evidence strength
Small primary tumour (cT1 to cT2) with multistation N2	No consensus after three Delphi rounds; representative borderline subset	Included within the stage III cohorts of AEGEAN, KEYNOTE-671, CheckMate 77T and RATIONALE-315; not analysed as a discrete subgroup (Yue et al., 2025a, Mitsudomi et al., 2025, Provencio et al., 2026)	Indirect
Larger primary tumour (cT3) with single-station N2	No consensus after three Delphi rounds; representative borderline subset	Included in all six pivotal trials; AEGEAN and CheckMate 77T report N2 subgroup analyses (Mitsudomi et al., 2025, Provencio et al., 2026, Garassino et al., 2024); RATIONALE-315 enrolled stage II to IIIA including N2 (Yue et al., 2025a)	Indirect (subgroup)
Invasive cT4 with maximum single-station N2	No consensus after three Delphi rounds; representative borderline subset	Small numbers within the pivotal trials; not separately reported	Sparse
Superior sulcus (Pancoast) tumours	Consensus generally resectable in the absence of N3, but individualised multidisciplinary decision	Excluded or under-represented in CheckMate 816 and AEGEAN	Very limited
Stage IIB to IIIB with prospectively multidisciplinary-defined borderline status	Population targeted by dedicated borderline resectable trials	MDT-BRIDGE (Reck et al., 2024) and NEOINSPIRE (ClinicalTrials.gov, 2026a) enrol this population prospectively	Direct (pending)

The AEGEAN N2 subgroup analysis (Garassino et al., 2024) is an ASCO 2024 conference abstract and has not been published in full. MDT-BRIDGE (Reck et al., 2024) and NEOINSPIRE (ClinicalTrials.gov, 2026a) are ongoing and have reported no efficacy data. Abbreviations: MDT, multidisciplinary team; N, nodal descriptor; T, tumour descriptor.

Functional operability runs orthogonally to that definition (Brunelli et al., 2009). A patient with cT3N1M0 disease and severely impaired pulmonary reserve is not borderline resectable; the tumour is resectable and the patient is marginal. The distinction matters for the argument developed in Section 3.4, because checkpoint inhibitor toxicity acts on the functional axis rather than the anatomical one. Pneumonitis, myocarditis, colitis with deconditioning and prolonged corticosteroid exposure all reduce physiological reserve without altering the T or N descriptor.

## 3 Current neoadjuvant and perioperative ICI in resectable NSCLC

### 3.1 Neoadjuvant-only regimens

CheckMate 816 established the neoadjuvant-only approach: three cycles of nivolumab with platinum doublet chemotherapy before resection, with no protocol checkpoint inhibitor afterwards, in 358 patients with resectable stage IB (4 cm or larger) to IIIA disease (Table 2) (Forde et al., 2022). Pathological complete response was 24.0% against 2.2% with chemotherapy alone, and event-free survival favoured the combination with a hazard ratio of 0.63 (95% CI 0.43–0.91) (Forde et al., 2022). At a median follow-up of 68.4 months, 5-year overall survival was 65.4% against 55.0% (Forde et al., 2025).

**TABLE 2:** Design, eligibility, efficacy and surgical outcome in the six pivotal phase 3 trials of neoadjuvant or perioperative PD-1 or PD-L1 blockade in resectable NSCLC. Values are experimental against control.

Trial (ref)	Regimen	Population and N	Key eligibility and molecular exclusions	pCR	EFS HR (95% CI)	R0	Definitive surgery	Additional outcome
CheckMate 816 (Forde et al., 2022), (Forde et al., 2025)	Neoadjuvant nivolumab plus platinum doublet, 3 cycles, no adjuvant	Resectable IB (4 cm or larger) to IIIA, N=358	AJCC 7th edition staging; ECOG 0 to 1; known sensitising *EGFR* mutation or *ALK* alteration excluded, testing not mandated in all regions	24.0% vs 2.2%	0.63 (0.43 to 0.91)	83.2% vs 77.8%	83.2% vs 75.4%	5-year OS 65.4% vs 55.0% at median follow-up 68.4 months
KEYNOTE-671 (Wakelee et al., 2023), (Spicer et al., 2024)	Perioperative pembrolizumab, 4 neoadjuvant plus up to 13 adjuvant cycles	Resectable II to IIIB(N2), N=797	AJCC 8th edition staging; ECOG 0 to 1; enrolment irrespective of PD-L1; *EGFR* and *ALK* alterations not excluded, status unknown in roughly two thirds	18.1% vs 4.0%	0.58 (0.46 to 0.72)	92.0% vs 84.2%	82.1% vs 79.4%	90-day mortality 4.0% vs 1.6%
AEGEAN (Heymach et al., 2023), (Spicer et al., 2024)	Perioperative durvalumab, 4 neoadjuvant plus up to 12 adjuvant cycles	Resectable IIA to IIIB(N2), N=802	AJCC 8th edition staging; ECOG 0 to 1; known *EGFR* or *ALK* alterations excluded from the modified ITT population by protocol amendment	17.2% vs 4.3%	0.68 (0.53 to 0.88)	94.7% vs 91.3%	Not reported in main text	N2 subgroup pCR 16.6% vs 4.9% (Garassino et al., 2024)
CheckMate 77T (Cascone et al., 2024), (Provencio et al., 2026)	Perioperative nivolumab, 4 neoadjuvant plus up to 13 adjuvant cycles	Resectable IIA (over 4 cm) to IIIB(N2), N=461	AJCC 8th edition staging; ECOG 0 to 1; *EGFR* mutations and known *ALK* translocations excluded, *EGFR* testing mandatory in nonsquamous disease	25.3% vs 4.7%	0.58 (0.42 to 0.81)	89% vs 90%	77.7% vs 76.7%	N2 pCR 22.0% vs 5.6%; nodal downstaging 67% vs 59% (Provencio et al., 2026)
NEOTORCH (Lu et al., 2024)	Perioperative toripalimab, 3 neoadjuvant plus 1 postoperative cycle then up to 13 adjuvant cycles	Resectable II to III; stage III set N=404	AJCC 8th edition staging; age 18 to 70; ECOG 0 to 1; *EGFR* and *ALK* alterations excluded in nonsquamous disease; China only	24.8% vs 1.0%	0.40 (0.28 to 0.57)	95.8% vs 92.6%	82.2% vs 73.3%	Fatal adverse events 3.0% vs 2.0%
RATIONALE-315 (Yue et al., 2025a), (Yue et al., 2025b)	Perioperative tislelizumab, 3 to 4 neoadjuvant cycles plus adjuvant	Resectable II to IIIA, N=453	AJCC 8th edition staging; ECOG 0 to 1; judged eligible for R0 resection at enrolment; known *EGFR* mutation (nonsquamous) or *ALK* translocation excluded; China only	40.7% vs 5.7%	0.56 (0.40 to 0.79)	95% vs 93%	84.1% vs 76.2%	Median time to surgery 13.4 vs 12.7 weeks

Pathological complete response definitions, central pathology review and eligibility windows differ between trials, so cross-trial comparison is descriptive only. Eligibility and molecular exclusion criteria are as stated in each primary publication or trial protocol; CheckMate 816 staged by AJCC 7th edition, all other trials by AJCC 8th edition. RATIONALE-315 values are from a prespecified interim analysis, and its R0 and definitive-surgery values are congress-reported (Yue et al., 2025b). NEOTORCH values are for the stage III set only. R0 rates are calculated among patients undergoing surgery. Abbreviations: EFS, event-free survival; HR, hazard ratio; OS, overall survival; pCR, pathological complete response.

Operative metrics did not deteriorate. Median operative time was 184 min with nivolumab against 217 min with chemotherapy alone, a minimally invasive approach was used in 30% against 22%, and conversion to open thoracotomy occurred in 11% against 16% (Forde et al., 2022; Forde et al., 2025). R0 resection was achieved in 83.2% against 77.8%, and 83.2% against 75.4% of randomised patients underwent definitive surgery (Forde et al., 2022). Grade 3 or 4 surgery-related adverse events were 11.4% against 14.8% (Forde et al., 2022).

Grade 3 or higher treatment-related adverse events were 33.5% against 36.9%, the lowest experimental-arm figure of the six pivotal trials (Table 3) (Forde et al., 2022). Treatment-related discontinuation was 10.2% against 9.7%, and delay to surgery attributed to an adverse event occurred in 3.4% against 5.1% (Forde et al., 2022). An aggregate immune-mediated adverse event rate was not reported, and the events that were described were mainly grade 1 to 2 (Forde et al., 2022). The trial therefore supports a narrow but useful claim: adding three cycles of PD-1 blockade to induction chemotherapy did not make the operation longer, more open or later, in a population selected as resectable at enrolment.

**TABLE 3:** Safety and surgical feasibility in the six pivotal phase 3 trials. Values are experimental against control.

Trial (ref)	Grade 3 or higher TRAE	Any-grade immune-mediated AE / grade 3 or higher	Pneumonitis	Discontinuation due to adverse event	Surgery not performed or cancelled	Delay or time to surgery	R0	Definitive surgery	Perioperative morbidity and mortality
CheckMate 816 (Forde et al., 2022), (Forde et al., 2025)	33.5% vs 36.9%	NR as an aggregate (described as low, mainly grade 1 to 2)	Any-grade 1.1%; grade 3 or higher 0% (control NR)	10.2% vs 9.7% (treatment-related)	Cancelled 15.6% vs 20.7%	Delay due to adverse event 3.4% vs 5.1%	83.2% vs 77.8%	83.2% vs 75.4%	Grade 3 or 4 surgery-related AEs 11.4% vs 14.8%
KEYNOTE-671 (Wakelee et al., 2023), (Spicer et al., 2024)	44.9% vs 37.3%	25.3% vs 10.5% / 5.8% vs 1.5%	Grade 3 or higher NR in main text	12.6% vs 5.3% (treatment-related, all study treatment)	Did not undergo surgery 17.9% vs 20.6%; unresectable 1.5% vs 4.7%	Median time to surgery NR in main text	92.0% vs 84.2%	82.1% vs 79.4%	Surgical-phase any AE 71.1% vs 71.3%; 30-day mortality 1.8% vs 0.6%; 90-day 4.0% vs 1.6%
AEGEAN (Heymach et al., 2023), (Mitsudomi et al., 2025)	42.4% vs 43.2%	25.4% vs 10.3% / 4.5% vs 2.5%	Grade 3 or higher approximately 3.0%	12.0% vs 6.0% (durvalumab or placebo)	AE-related cancellation 1.7% vs 1.0%	Any delay 17.3% vs 22.2%	94.7% vs 91.3%	NR in main text	NR by arm in main text
NEOTORCH (Lu et al., 2024)	63.4% vs 54.0%	42.1% vs 22.8% / 11.9% vs 3.0%	NR	9.4% vs 7.4% (treatment-emergent)	NR	NR	95.8% vs 92.6%	82.2% vs 73.3%	Fatal adverse events 3.0% vs 2.0%
CheckMate 77T (Cascone et al., 2024), (Provencio et al., 2026)	32.5% vs 25.2%	Described as infrequent, mostly grade 1 to 2; grade 3 or higher NR	Two treatment-related deaths from pneumonitis in the experimental arm	19.3% vs 7.4% (treatment-related)	NR in main text	NR	NR in main text	77.7% vs 76.7%	Surgery-related AEs 41.0% vs 38.8%
RATIONALE-315 (Yue et al., 2025a), (Yue et al., 2025b)	73.0% vs 67.3%	40.3% vs 18.1% / 9.3% vs 3.1%	One pneumonitis death in the tislelizumab arm (0.4%)	12.8% vs 9.3%	Cancellation for any reason 15.9% vs 23.8%; treatment-related 0.4% vs 0.4%	Treatment-related delay 5.3% vs 1.8%; median time to surgery 13.4 vs 12.7 weeks	95% vs 93%	84.1% vs 76.2%	NR

Values are experimental against control, taken from the primary publications, their surgical-outcomes companion papers and, for RATIONALE-315, congress presentations (Yue et al., 2025b). Immune-toxicity terminology is trial-specific, immune-mediated adverse events in the AEGEAN, KEYNOTE-671 and CheckMate programmes and immune-related adverse events in NEOTORCH and RATIONALE-315, and the rates are therefore not directly comparable. Discontinuation figures are treatment-related unless stated otherwise. The AEGEAN grade 3 or higher figure of 42.4% against 43.2% derives from an all-cause grade 3 or 4 adverse event report rather than from a treatment-related table. Abbreviations: AE, adverse event; NR, not reported; TRAE, treatment-related adverse event.

### 3.2 Perioperative regimens

Five phase 3 trials continued checkpoint inhibition after resection. KEYNOTE-671 gave four neoadjuvant cycles of pembrolizumab with chemotherapy followed by up to 13 adjuvant cycles in 797 patients with stage II to IIIB(N2) disease, reporting pCR of 18.1% against 4.0% and EFS hazard ratio 0.58 (95% CI 0.46–0.72) (Wakelee et al., 2023; Spicer et al., 2024). AEGEAN used durvalumab with four neoadjuvant and up to 12 adjuvant cycles in 802 patients with stage IIA to IIIB(N2) disease, reporting pCR of 17.2% against 4.3% and hazard ratio 0.68 (95% CI 0.53–0.88) (Heymach et al., 2023). CheckMate 77T reported pCR of 25.3% against 4.7% and hazard ratio 0.58 (95% CI 0.42–0.81) among 461 patients with stage IIA to IIIB disease (Cascone et al., 2024). NEOTORCH, in its stage III set of 404 patients, reported pCR of 24.8% against 1.0% and the largest effect estimate of the group, hazard ratio 0.40 (95% CI 0.28–0.57) (Lu et al., 2024).

RATIONALE-315 is new to this appraisal and is the sixth pivotal trial. Perioperative tislelizumab with three to four neoadjuvant chemotherapy cycles in 453 patients with resectable stage II to IIIA disease produced pCR of 40.7% against 5.7% and EFS hazard ratio 0.56 (95% CI 0.40–0.79) at a prespecified interim analysis (Yue et al., 2025a). That pCR is the highest reported in a phase 3 trial in resectable NSCLC, and it comes from an interim rather than a final analysis (Yue et al., 2025a). R0 resection of 95% against 93% and definitive surgery in 84.1% against 76.2% are congress-reported rather than published in the primary interim paper (Yue et al., 2025b).

The adjuvant phase is where perioperative regimens shed patients. Treatment-related discontinuation of all study treatment reached 19.3% against 7.4% in CheckMate 77T and 12.6% against 5.3% in KEYNOTE-671 (Wakelee et al., 2023; Cascone et al., 2024), and 9.4% against 7.4% of patients in NEOTORCH discontinued for a treatment-emergent event (Lu et al., 2024). What proportion of the survival benefit the adjuvant component contributes remains untested, because no trial randomised the postoperative phase separately. Durvalumab nonetheless holds regulatory approval for perioperative use in resectable NSCLC, so the question is now being answered in practice rather than in trials (US Food and Drug Administration, 2024).

### 3.3 Neoadjuvant vs. perioperative comparison

No trial has randomised patients between a neoadjuvant-only and a perioperative strategy. The available comparison is indirect and meta-analytic: across randomised trials in resectable NSCLC, neoadjuvant and perioperative administration were each associated with greater overall survival benefit than adjuvant-only administration, with the two preoperative strategies not separable from one another (Patel et al., 2025). We do not quote hazard ratios from that analysis, because the corresponding 95% confidence intervals are not reported in the data available to us.

Cross-trial toxicity figures invite a comparison they cannot sustain. Grade 3 or higher treatment-related adverse events were 33.5% in the CheckMate 816 experimental arm, 63.4% in NEOTORCH and 73.0% in RATIONALE-315 (Forde et al., 2022; Lu et al., 2024; Yue et al., 2025a). Reading that gradient as the price of the adjuvant phase would be wrong for three reasons. First, the chemotherapy backbones, cycle numbers and geographies differ across the three trials. Second, the control arms move in the same direction, at 36.9%, 54.0% and 67.3% respectively, which points to population characteristics and reporting conventions rather than to the checkpoint inhibitor (Forde et al., 2022; Lu et al., 2024; Yue et al., 2025a). Third, the AEGEAN figure of 42.4% derives from an all-cause grade 3 or 4 report rather than from a treatment-related table, so the column is not internally consistent across trials (Heymach et al., 2023).

For the operating surgeon the practical difference between the strategies is not resectability but the timing of exposure. A neoadjuvant-only regimen closes immune exposure at the point of resection, so the postoperative course is managed without concurrent checkpoint inhibition. A perioperative regimen adds up to a year of exposure after the decision to operate has been taken and executed. That bears on the management of prolonged air leak, empyema, reoperation and pulmonary rehabilitation, and on the interpretation of new breathlessness in a patient with one fewer lobe, rather than on whether the tumour comes out.

Two designs would resolve the question. A randomised comparison of continuing against stopping checkpoint inhibition after resection, stratified by pathological response, would isolate the adjuvant contribution. A response-adapted design, in which patients achieving pCR stop and those with residual viable tumour continue, would test the same question against a plausible biological hypothesis and would use the surrogate that CheckMate 816 and its pooled analyses have supported (Forde et al., 2025; Deutsch et al., 2024). Neither approach is currently reported in the trials reviewed here.

### 3.4 Immune-related toxicity and surgical scheduling

Immune-mediated adverse events cluster in the induction window, which is the same window in which operability is reassessed. KEYNOTE-671 reported any-grade immune-mediated events in 25.3% against 10.5%, with grade 3 or higher in 5.8% against 1.5% (Wakelee et al., 2023). AEGEAN reported 25.4% against 10.3%, with grade 3 or higher in 4.5% against 2.5% (Heymach et al., 2023). NEOTORCH reported immune-related events in 42.1% against 22.8%, with grade 3 or higher in 11.9% against 3.0% (Lu et al., 2024), and RATIONALE-315 reported 40.3% against 18.1%, with grade 3 or higher in 9.3% against 3.1% (Yue et al., 2025a). These rates are not comparable with one another. The trials use different terminology, different adjudication and different reporting windows, and CheckMate 816 and CheckMate 77T did not report an aggregate figure at all (Forde et al., 2022; Cascone et al., 2024).

Pneumonitis is the event most likely to remove surgical candidacy outright, and it is uncommon. CheckMate 816 reported any-grade pneumonitis in 1.1% of the experimental arm with no grade 3 or higher events, the control-arm rate not being reported (Forde et al., 2022). AEGEAN reported grade 3 or higher pneumonitis in approximately 3.0% (Heymach et al., 2023). Two treatment-related deaths from pneumonitis occurred in the experimental arm of CheckMate 77T (Cascone et al., 2024), and one pneumonitis death, 0.4% of the tislelizumab arm, occurred in RATIONALE-315 (Yue et al., 2025a). A patient with grade 2 pneumonitis on corticosteroids is not a candidate for lobectomy until the infiltrate and the steroid have resolved, and in a borderline case that interval is often the interval in which the window for resection closes.

Cancellation and delay must be separated. Treatment-related cancellation of surgery was 1.7% against 1.0% in AEGEAN (Mitsudomi et al., 2025) and 0.4% against 0.4% in RATIONALE-315 (Yue et al., 2025a). All-cause non-receipt of surgery was an order of magnitude higher, at 15.6% against 20.7% in CheckMate 816% and 15.9% against 23.8% in RATIONALE-315 (Forde et al., 2022; Yue et al., 2025a), with disease progression, patient choice and intraoperative findings of unresectability accounting for most of the difference; KEYNOTE-671 recorded unresectable disease in 1.5% against 4.7% (Wakelee et al., 2023). The same asymmetry holds for delay. Any delay to surgery occurred in 17.3% against 22.2% in AEGEAN (Mitsudomi et al., 2025), whereas treatment-related delay in RATIONALE-315 was 5.3% against 1.8%, and median time from randomisation to surgery was 13.4 against 12.7 weeks (Yue et al., 2025a). Toxicity contributes a small fraction of both cancellation and delay, and in the control arms cancellation and delay were often more frequent, not less.

Perioperative outcome after chemoimmunotherapy has been reported in detail by only one of the six trials. KEYNOTE-671 recorded 30-day mortality of 1.8% against 0.6% and 90-day mortality of 4.0% against 1.6%, with any surgical-phase adverse event in 71.1% against 71.3% (Wakelee et al., 2023). CheckMate 816 recorded grade 3 or 4 surgery-related events in 11.4% against 14.8% (Forde et al., 2022), and CheckMate 77T recorded surgery-related events in 41.0% against 38.8% (Cascone et al., 2024). Technical descriptions of post-immunotherapy hilar fibrosis and nodal adherence come from a small retrospective series that predates the pivotal trials and reports no measurable outcome penalty (Bott et al., 2018). The honest summary is that dissection may be harder in some patients and that no trial has quantified it.

We propose an operational rule, and label it as opinion rather than evidence. Any active grade 2 or higher immune-mediated event should be resolved, or stable on physiological-replacement corticosteroid alone, before thoracotomy or minimally invasive lobectomy, with a defined maximum interval from the last induction cycle agreed at the multidisciplinary meeting rather than negotiated case by case. The risk envelope for that decision is the ASCO threshold for offering surgery in stage III disease, which requires R0 resection to be judged achievable and perioperative 90-day mortality not to exceed approximately 5% (Daly et al., 2022). The observed 90-day mortality of 4.0% in KEYNOTE-671 sits inside that envelope but leaves little room in a patient whose baseline reserve is already marginal (Wakelee et al., 2023).

## 4 Emerging targets, ADCs and bispecifics

The appraisal question for every emerging agent is whether it earns preoperative exposure in a patient who could be operated on today (Table 4). That question is stricter than the question asked in advanced disease, because the alternative is not a different systemic regimen but an operation with curative intent that is already available. We apply three tests to each class. First, is there a positive randomised phase 3 read-out in advanced disease, since a target that fails there has no mechanistic claim on the preoperative window. Second, is there a dedicated neoadjuvant signal, meaning pathological response and surgical feasibility data from patients taken to theatre rather than extrapolation from a metastatic cohort. Third, how does the therapeutic index compare with PD-1 chemoimmunotherapy, taking as the reference point the NEOSTAR observation that adding CTLA-4 blockade to nivolumab raised major pathological response from 22% to 38% while resection was completed in 16 of 21 against 21 of 23 patients (Cascone et al., 2021). Response gains that cost operations are not gains in this population.

**TABLE 4:** Emerging targets in resectable NSCLC by evidence level, peer-review status, key efficacy estimate and toxicity of preoperative relevance.

Target and axis	Agent	Highest evidence level and peer-review status	Key efficacy figure (95% CI where reported)	Toxicity of preoperative relevance	Neoadjuvant data
TIGIT, co-inhibitory	Tiragolumab	Phase 3 negative in three settings; peer-reviewed (Peters et al., 2026, Rudin et al., 2024, Wakelee et al., 2026)	SKYSCRAPER-01 OS HR 0.87 (0.70 to 1.10), p=0.22	Grade 3 to 4 AEs 41.2% vs 33.8% (Peters et al., 2026)	No
TIGIT, bispecific format	Rilvegostomig (PD-1/TIGIT)	Phase 1/2 conference presentation only (Hiltermann et al., 2024), ClinicalTrials.gov, 2026e)	Confirmed plus pending ORR 61.8% at 750 mg in TPS 50% or higher	Grade 3 or higher immune-mediated AEs 2.1% (Hiltermann et al., 2024)	No (NeoCOAST-2 Arms 6 and 7 not reported)
LAG-3, co-inhibitory	Relatlimab	Randomised phase 2; peer-reviewed (Schuler et al., 2024)	pCR 17% vs 13%; mPR 30% vs 27%	Grade 3 or higher TRAEs 13% vs 10% (Schuler et al., 2024)	Yes
TIM-3, co-inhibitory	Cobolimab	Phase 3 negative; company-reported only, no peer-reviewed publication (ClinicalTrials.gov, 2026b), (GSK plc, 2025)	No OS improvement over docetaxel	Not reported	No
NKG2A, immune-metabolic	Monalizumab	Platform phase 2; peer-reviewed (Cascone et al., 2023), ((Cascone et al., 2025))	pCR 25.7% (16.0 to 37.6); mPR 50.0% (37.8 to 62.2)	Grade 3 or higher TRAEs 40.8%; arm-level grade 3 or higher irAEs NR (Cascone et al., 2025)	Yes
CD73, immune-metabolic	Oleclumab	Platform phase 2; peer-reviewed (Cascone et al., 2023), (Cascone et al., 2025)	pCR 20.3% (11.8 to 31.2); mPR 41.9% (30.5 to 53.9)	Grade 3 or higher TRAEs 36.5%; arm-level grade 3 or higher irAEs NR (Cascone et al., 2025)	Yes
TROP2, antibody-drug conjugate	Datopotamab deruxtecan	Platform phase 2; peer-reviewed (Cascone et al., 2025)	pCR 35.2% (22.7 to 49.4); mPR 63.0% (48.7 to 75.7)	Oral mucositis or stomatitis 40.7%; ocular-surface events 20.4%; one grade 2 drug-related ILD (Cascone et al., 2025)	Yes
B7-H3, tumour-associated	Enoblituzumab	Phase 1/2; peer-reviewed (Aggarwal et al., 2022)	ORR 35.7% (12.8 to 64.9) in 14 patients	Grade 3 or higher TRAEs 28.6%; one pneumonitis death (Aggarwal et al., 2022)	No
Siglec-15, myeloid	NC318	Phase 1 and phase 2 conference abstracts (Patnaik et al., 2021), (Gettinger et al., 2023)	Durable clinical benefit 28% (preliminary)	Not reported in detail	No
ICOS, costimulatory	Vopratelimab, feladilimab	Phase 1/2; peer-reviewed (Yap et al., 2022), (Maio et al., 2025)	ORR 1.4% monotherapy; 2.3% with nivolumab (Yap et al., 2022)	Tolerable; NSCLC sub-study closed for futility (Maio et al., 2025)	No
PD-1 and VEGF, bispecific	Ivonescimab	Phase 3 positive; peer-reviewed (Xiong et al., 2025)	Median PFS 11.1 vs 5.8 months, HR 0.51 (0.38 to 0.69), p<0.001	Grade 3 or higher TRAEs 29% vs 16% (Xiong et al., 2025)	No (NEOINSPIRE ongoing (ClinicalTrials.gov, 2026a))
PD-1 and CTLA-4, bispecific	Volrustomig	Phase 1 in mixed solid tumours, peer-reviewed (Tran et al., 2026); NSCLC data abstract only (Spigel et al., 2024)	ORR 19.8% in 86 patients with mixed tumours	Immune-endocrine toxicity; one treatment-related death (Tran et al., 2026)	No (NeoCOAST-2 Arm 3 not reported)
PD-L1 and VEGF-A, bispecific	Pumitamig	Conference abstract and company announcement (BioNTech and Myers Squibb, 2026)	Confirmed ORR 57.1% nonsquamous; 68.4% squamous	Grade 3 or higher TRAEs 48.8%; bleeding events 20.9% (BioNTech and Myers Squibb, 2026)	No

Peer-review status is stated for every row because four programmes rest on conference abstracts or company statements [(Gettinger et al., 2023), (Spigel et al., 2024), (BioNTech and Myers Squibb, 2026), (Hiltermann et al., 2024). Datopotamab deruxtecan is TROP2-directed, not B7-H3-directed. Abbreviations: AE, adverse event; HR, hazard ratio; ILD, interstitial lung disease; irAE, immune-related adverse event; mPR, major pathological response; NR, not reported; ORR, objective response rate; OS, overall survival; pCR, pathological complete response; PFS, progression-free survival; TPS, tumour proportion score; TRAE, treatment-related adverse event.

### 4.1 Co-inhibitory checkpoints (TIGIT, LAG-3, TIM-3)

TIGIT was the most heavily invested co-inhibitory target after PD-1, and it has failed. The randomised phase 2 CITYSCAPE study of tiragolumab plus atezolizumab in PD-L1-selected metastatic NSCLC reported an objective response rate of 31.3% against 16.2% in 67 and 68 patients respectively, with median progression-free survival of 5.4 against 3.6 months, hazard ratio 0.57 (Cho et al., 2022). Serious treatment-related events occurred in 21% against 18%, with two treatment-related deaths in the combination arm (Cho et al., 2022). That signal did not replicate. SKYSCRAPER-01 randomised 521 patients with PD-L1-high locally advanced or metastatic disease. Investigator-assessed median progression-free survival was 7.0 against 5.6 months, hazard ratio 0.78 (95% CI 0.63–0.97), with a nominal p = 0.02 that did not cross the prespecified multiplicity boundary. Final median overall survival was 23.1 against 16.9 months, hazard ratio 0.87 (95% CI 0.70–1.10), p = 0.22, with grade 3 to 4 adverse events in 41.2% against 33.8% and four against two treatment-related deaths (Peters et al., 2026).

The two companion trials were also negative. SKYSCRAPER-02, in untreated extensive-stage small cell lung cancer, reported median progression-free survival of 5.4 against 5.6 months, hazard ratio 1.11, p = 0.35, and median overall survival of 13.1 against 13.1 months, hazard ratio 1.14, p = 0.29, with grade 3 to 4 immune-mediated events in 7.9% against 7.7% (Rudin et al., 2024). SKYSCRAPER-03 tested tiragolumab plus atezolizumab against durvalumab consolidation after chemoradiation in unresectable stage III disease and missed its primary endpoint. PD-L1-positive median progression-free survival was 19.4 against 16.6 months, hazard ratio 0.96 (95% CI 0.75–1.23), p = 0.76, with an all-comers hazard ratio of 1.00 (95% CI 0.84–1.19) and overall survival hazard ratios of 0.99 and 0.98 (Wakelee et al., 2026). Three randomised settings, three failures, including the setting anatomically closest to borderline resectable disease. Monospecific TIGIT blockade cannot be advanced into a preoperative regimen on this evidence. Whatever residual rationale exists lies in a bispecific format that couples TIGIT to PD-1 within one molecule (Hiltermann et al., 2024), or in a biomarker-defined subset that has not yet been defined.

LAG-3 has the opposite profile: a small neoadjuvant signal and no randomised phase 3 test in NSCLC. Dual LAG-3 and PD-1 blockade improved progression-free survival over nivolumab in untreated advanced melanoma, which is the precedent (Tawbi et al., 2022). NEOpredict-Lung randomised 60 patients with resectable stage IB to IIIA disease to neoadjuvant nivolumab with or without relatlimab. The combination produced pCR of 17% and major pathological response of 30%, against 13% and 27% with nivolumab alone, with surgery in 28 of 30 against 30 of 30 patients and grade 3 or higher treatment-related events in 13% against 10% (Schuler et al., 2024). The trial was not powered for an efficacy comparison, the differences are within sampling noise at this sample size, and the population was favourable rather than borderline.

TIM-3 has been tested and abandoned. The receptor is co-expressed with PD-1 on exhausted tumour-infiltrating lymphocytes and was a coherent second target (Acharya et al., 2020). In COSTAR Lung, neither the cobolimab, dostarlimab and docetaxel triplet nor the dostarlimab plus docetaxel doublet improved overall survival over docetaxel, and the programme was discontinued (ClinicalTrials.gov, 2026b; GSK plc, 2025). No peer-reviewed phase 3 publication exists, and the evidence is company-reported through a registry record and a quarterly results announcement, which is a weaker basis than we would accept for a positive claim and which we apply symmetrically here. BTLA and VISTA remain preclinical or early phase 1 in NSCLC with no neoadjuvant data and no effect estimates that could be quoted (Demerle et al., 2021; ElTanbouly et al., 2020).

### 4.2 Immune-metabolic pathways (NKG2A, CD73)

NKG2A and CD73 target the inhibitory and adenosine-mediated arms of the tumour microenvironment rather than the T cell synapse directly (Allard et al., 2020; Inoue et al., 2017). In the COAST platform trial of consolidation therapy after chemoradiation in unresectable stage III disease, 66 patients received durvalumab, 59 received durvalumab plus the anti-CD73 antibody oleclumab and 61 received durvalumab plus the anti-NKG2A antibody monalizumab, with objective response rates of 23.9%, 35.0% and 40.3% (Herbst et al., 2022; Aggarwal et al., 2025). Progression-free survival favoured both combinations, hazard ratio 0.59 (95% CI 0.37–0.93) for oleclumab and 0.63 (95% CI 0.40–0.99) for monalizumab, but the overall survival estimates of 0.69 (95% CI 0.40–1.20) and 0.77 (95% CI 0.44–1.33) both cross 1, so no survival benefit was established (Aggarwal et al., 2025). PACIFIC-9 is the phase 3 test of both combinations in the same setting and has reported no efficacy data; the available publication is a trial design paper (Barlesi et al., 2024).

The neoadjuvant signals come from the NeoCOAST platform. The original NeoCOAST trial gave single-cycle neoadjuvant durvalumab alone or combined with oleclumab, monalizumab or danvatirsen in arms of 27, 21, 20 and 16 patients. Major pathological response was 11.1%, 19.0%, 30.0% and 31.3%, pCR was 3.7%, 9.5%, 10.0% and 12.5%, and grade 3 or higher treatment-related events were 0%, 4.8%, 0% and 6.3% (Cascone et al., 2023). The arms are too small for formal comparison and no comparison was prespecified.

NeoCOAST-2 gave four cycles of durvalumab with chemotherapy plus a partner agent. In the monalizumab arm, pCR was 18 of 70, 25.7% (95% CI 16.0–37.6), and major pathological response was 35 of 70, 50.0% (95% CI 37.8–62.2) (Cascone et al., 2025). Surgery was performed in 66 of 71 patients, 93.0%, with R0 in 92.4%, grade 3 or higher treatment-related events in 40.8%, and any-grade immune-mediated events during neoadjuvant treatment in 12.7% (Cascone et al., 2025). In the oleclumab arm, pCR was 15 of 74, 20.3% (95% CI 11.8–31.2), major pathological response 31 of 74, 41.9% (95% CI 30.5–53.9), surgery 93.2%, R0 91.3%, grade 3 or higher treatment-related events 36.5%, and any-grade immune-mediated events 6.8% (Cascone et al., 2025). One treatment-related death occurred in each arm, from colonic obstruction and from intestinal ischaemia with septic shock respectively (Cascone et al., 2025). Arm-level grade 3 or higher immune-mediated event rates were not reported for either arm, and we do not substitute the durvalumab-attributed grade 3 or higher figures in their place (Cascone et al., 2025). High CD73 expression is associated with worse prognosis in resected NSCLC, which is a prognostic observation and not predictive validation of an anti-CD73 antibody (Inoue et al., 2017); first-in-human data for oleclumab alone and with durvalumab establish tolerability rather than activity in this setting (Bendell et al., 2023).

### 4.3 TROP2-directed antibody-drug conjugates

Datopotamab deruxtecan is a TROP2-directed antibody-drug conjugate with a cleavable linker and a deruxtecan topoisomerase-I inhibitor payload (Cascone et al., 2025). It is not a B7-H3-directed agent, and the NeoCOAST-2 signal described below belongs to the TROP2 axis rather than to the costimulatory or B7-family targets discussed in Section 4.4. We state this explicitly because the attribution was made incorrectly in the previous version of this review.

In NeoCOAST-2 Arm 4, datopotamab deruxtecan with durvalumab and carboplatin produced pCR in 19 of 54 patients, 35.2% (95% CI 22.7–49.4), and major pathological response in 34 of 54, 63.0% (95% CI 48.7–75.7) (Cascone et al., 2025). Surgery was performed in 51 of 54 patients, 94.4%, and R0 resection was achieved in 88.0% of the 50 completed operations, the denominator reflecting one attempted sleeve lobectomy that could not be completed because of scar tissue (Cascone et al., 2025). Grade 3 or higher treatment-related events occurred in 11 of 54 patients, 20.4%, the lowest of the three reported arms (Cascone et al., 2025).

The toxicity profile is not an immune profile, and its preoperative relevance is different in kind. Oral mucositis or stomatitis affected 40.7% of patients, ocular-surface events 20.4% and mucosal inflammation 14.8%, and there was one grade 2 adjudicated drug-related interstitial lung disease event, 1 of 54 patients (Cascone et al., 2025). Surgery was delayed beyond 40 days in 27.5%, mostly for logistical reasons (Cascone et al., 2025). Mucositis and ocular surface disease do not remove operability, but they degrade nutrition, oral intake and prehabilitation in the weeks before a thoracotomy, which is precisely where a borderline patient has least reserve. One treatment-related death occurred, from postoperative interstitial lung disease that the investigator assessed as unrelated and adjudication assessed as related (Cascone et al., 2025).

Calibration matters here. A pCR of 35.2% sits within, not above, the range established by the pivotal phase 3 trials, because RATIONALE-315 reported 40.7% (Yue et al., 2025a; Cascone et al., 2025). Arm 4 is a single-arm cohort of 54 patients within a platform trial, without a randomised comparator and without a borderline resectable subgroup. Arms 3 and 5 to 7 of the platform, covering volrustomig, the anti-LIF antibody AZD0171 and rilvegostomig with or without datopotamab deruxtecan, have not reported arm-level results (Cascone et al., 2025).

### 4.4 Costimulatory and B7-family agents

B7-H3 is overexpressed in NSCLC with limited normal-tissue expression, which makes it attractive for both antibody and conjugate formats (Picarda et al., 2016). Clinical data remain early. Enoblituzumab plus pembrolizumab produced responses in 5 of 14 checkpoint-naive, PD-L1-negative patients with NSCLC, an objective response rate of 35.7% (95% CI 12.8–64.9), and across all 133 patients treated in the trial grade 3 or higher treatment-related events occurred in 28.6% with one treatment-related death from pneumonitis (Aggarwal et al., 2022). A confidence interval spanning 12.8%–64.9% cannot support a preoperative decision.

Costimulatory agonism has repeatedly under-delivered. The ICOS agonist vopratelimab produced objective response rates of 1.4% as monotherapy and 2.3% with nivolumab in the ICONIC trial (Yap et al., 2022), and the first-in-human study of the ICOS agonist feladilimab found the agent tolerable with limited monotherapy activity, its NSCLC sub-study closing for futility (Maio et al., 2025). High OX40 expression in the immune infiltrate is a favourable prognostic factor in NSCLC, and the OX40 agonist MOXR0916 was tolerable with limited single-agent activity (Massarelli et al., 2019; Diab et al., 2022). GITR, 4-1BB and CD40 agonist programmes have generated no NSCLC neoadjuvant signal (Mayes et al., 2018).

Siglec-15 is mechanistically distinct and is expressed in approximately 25% of NSCLC samples, largely mutually exclusively with PD-L1 (Wang et al., 2019). In a first-in-human phase 1 study, the anti-Siglec-15 antibody NC318 produced two objective responses and three cases of stable disease among 10 PD-1-refractory patients with NSCLC (Patnaik et al., 2021), and a phase 2 study reported durable clinical benefit in 28% of patients, presented at WCLC 2023 and available as a conference abstract only (Gettinger et al., 2023). The programme continues in combination with pembrolizumab (ClinicalTrials.gov, 2023). The mutually exclusive expression pattern is the interesting part, because it identifies a population that PD-1 blockade does not serve, but nothing in this dataset supports preoperative use.

### 4.5 Bispecific antibodies (PD-1/VEGF, PD-1/CTLA-4, PD-1/TIGIT, PD-l1/VEGF)

Ivonescimab, a tetravalent PD-1 and VEGF bispecific, is the only agent in this section with a positive peer-reviewed phase 3 result. In HARMONi-2, 398 patients with PD-L1-positive advanced NSCLC were randomised to ivonescimab or pembrolizumab. Median progression-free survival was 11.1 against 5.8 months, hazard ratio 0.51 (95% CI 0.38–0.69), p < 0.001, with grade 3 or higher treatment-related events in 29% against 16% and grade 3 or higher immune-related events in 7% against 8% (Xiong et al., 2025). Two qualifications belong with that result: the trial was conducted entirely in China, and overall survival was immature at the reported analysis (Xiong et al., 2025). NEOINSPIRE will provide the first randomised preoperative comparison of a PD-1 and VEGF bispecific against a PD-1 antibody, and has reported nothing (ClinicalTrials.gov, 2026a).

Volrustomig, a PD-1 and CTLA-4 bispecific, has peer-reviewed data only from a first-in-human study of 86 patients with mixed advanced solid tumours, in which the objective response rate was 19.8% with substantial immune-endocrine toxicity and one treatment-related death (Tran et al., 2026). Those data are not an NSCLC chemotherapy dataset and should not be read as one. NSCLC data with platinum doublet chemotherapy exist as a WCLC 2024 conference abstract (Spigel et al., 2024), and NeoCOAST-2 Arm 3, which tests volrustomig with chemotherapy preoperatively, has not reported (Cascone et al., 2025).

Pumitamig, a PD-L1 and VEGF-A bispecific, was reported in 40 response-evaluable patients, with confirmed objective response rates of 57.1% in nonsquamous and 68.4% in squamous disease (BioNTech and Myers Squibb, 2026). Grade 3 or higher treatment-related events occurred in 48.8%, grade 3 or higher immune-related events in 4.7%, bleeding events in 20.9% including one grade 3 event, and treatment-related discontinuation in 9.3% (BioNTech and Myers Squibb, 2026). These data come from a conference abstract and a company announcement, and phase 3 programmes are ongoing (ClinicalTrials.gov, 2026c; ClinicalTrials.gov, 2026d). A bleeding rate of 20.9% is the figure a surgeon should read first in an agent proposed for the weeks before a mediastinal dissection.

Rilvegostomig is a PD-1 and TIGIT bispecific. Combined confirmed and pending response rates were 29.0%, 9 of 31 patients, at 750 mg in PD-L1 tumour proportion score 1%–49%, and 61.8%, 21 of 34, at 750 mg in tumour proportion score 50% or higher (Hiltermann et al., 2024). There were no grade 4 to 5 treatment-related events, grade 3 or higher immune-mediated events occurred in 2.1% and treatment-related discontinuation in 4.2% (Hiltermann et al., 2024; ClinicalTrials.gov, 2026e). Combined confirmed and pending responses are not a confirmed objective response rate, and the data are a conference presentation. NeoCOAST-2 Arms 6 and 7, which test rilvegostomig with chemotherapy with or without datopotamab deruxtecan, have not reported (Cascone et al., 2025).

The evidence hierarchy across these four agents should be stated rather than implied. One bispecific has a peer-reviewed positive phase 3 trial in advanced disease (Xiong et al., 2025), one has a peer-reviewed phase 1 study in non-NSCLC populations (Tran et al., 2026), and two rest on conference abstracts or company statements (BioNTech and Myers Squibb, 2026; Hiltermann et al., 2024). None has reported neoadjuvant pathological response or surgical feasibility data. Against that, the NEOSTAR comparison of nivolumab plus ipilimumab against nivolumab showed what dual checkpoint blockade costs preoperatively, with major pathological response of 38% against 22% and pCR of 38% against 10%, but resection completed in 16 of 21 against 21 of 23 patients (Cascone et al., 2021). A bispecific format may improve on that trade-off by restricting dual engagement to the tumour microenvironment; no preoperative dataset yet shows that it does.

### 4.6 Biomarker-informed stratification

Applying the three tests set out at the start of this section places the emerging landscape into five tiers. Monospecific TIGIT blockade and TIM-3 blockade occupy the first tier, negative in randomised testing in advanced disease with no neoadjuvant case to make (Cho et al., 2022; Peters et al., 2026; Rudin et al., 2024; Wakelee et al., 2026; ClinicalTrials.gov, 2026b; GSK plc, 2025). The second tier holds agents with an unconfirmed neoadjuvant signal and an acceptable therapeutic index: LAG-3 in a 60-patient randomised phase 2 trial (Schuler et al., 2024), and NKG2A and CD73 across COAST, NeoCOAST and NeoCOAST-2 (Herbst et al., 2022; Aggarwal et al., 2025; Cascone et al., 2023; Cascone et al., 2025). The third tier contains one agent, the TROP2 antibody-drug conjugate, which has a neoadjuvant signal with preserved surgical feasibility but a non-immune toxicity profile whose preoperative consequences are nutritional and mucosal rather than inflammatory (Cascone et al., 2025). The fourth tier holds ivonescimab, positive in phase 3 in advanced disease with no neoadjuvant data at all (Xiong et al., 2025). The fifth tier is everything resting on abstracts, company statements, phase 1 studies or preclinical work: volrustomig (Tran et al., 2026; Spigel et al., 2024), pumitamig (BioNTech and Myers Squibb, 2026), rilvegostomig (Hiltermann et al., 2024), B7-H3 (Picarda et al., 2016; Aggarwal et al., 2022), Siglec-15 (Wang et al., 2019; Patnaik et al., 2021; Gettinger et al., 2023), BTLA (Demerle et al., 2021), VISTA (ElTanbouly et al., 2020) and the costimulatory agonists (Massarelli et al., 2019; Diab et al., 2022; Yap et al., 2022; Maio et al., 2025; Mayes et al., 2018).

Target-specific biomarkers would be needed to move any tier-two or tier-three agent into a borderline resectable population, and none is validated. TROP2 expression, CD73 expression, Siglec-15 expression and B7-H3 H-score are all measurable and none has been shown to predict differential benefit (Cascone et al., 2025; Aggarwal et al., 2022; Wang et al., 2019). That is a different situation from PD-1 blockade, where PD-L1 expression at least stratifies effect size across large randomised datasets.

No emerging target currently carries evidence sufficient to displace PD-1 chemoimmunotherapy in the preoperative treatment of borderline resectable disease. The strongest candidates for a randomised preoperative test, on the evidence reviewed here, are the NKG2A and TROP2 combinations from the NeoCOAST-2 platform (Cascone et al., 2025) and ivonescimab, which has the phase 3 result but no surgical data (ClinicalTrials.gov, 2026a; Xiong et al., 2025).

## 5 Implications for borderline resectable disease

### 5.1 The missing infrastructure is a prospective definition

Every safety figure quoted in Sections 3, 4 describes a population that was judged resectable before treatment began. None describes the population in which the resectability judgement was contested. Two trials are attempting to close that gap. MDT-BRIDGE will enrol approximately 140 patients with stage IIB to IIIB(N2), EGFR and ALK wild-type disease, give two cycles of neoadjuvant durvalumab with chemotherapy, and then reassess at a multidisciplinary meeting, with resection rate as the primary endpoint and primary completion anticipated in 2026 (Reck et al., 2024). NEOINSPIRE will randomise ivonescimab with chemotherapy against penpulimab with chemotherapy preoperatively (ClinicalTrials.gov, 2026a). MDT-BRIDGE is a published design paper and NEOINSPIRE is a registry entry; neither has reported any outcome.

### 5.2 Pathological downstaging is not conversion to resectable

Trial N2 subgroup analyses are frequently read as evidence that induction chemoimmunotherapy converts borderline cases to resectable ones. They do not show that. In the AEGEAN N2 subgroup, reported as an ASCO 2024 abstract, pCR was 16.6% against 4.9% and major pathological response 32.6% against 15.1% (Garassino et al., 2024). In the CheckMate 77T exploratory nodal analysis, N2 pCR was 22.0% against 5.6% with nodal downstaging in 67% against 59% (Provencio et al., 2026). Every one of those proportions is calculated among patients who reached surgery, in trials that had already selected patients as resectable (Mitsudomi et al., 2025; Provencio et al., 2026). Pathological downstaging is a treatment-effect measure in operated patients; conversion is a change in a multidisciplinary decision. No pivotal trial measured the second, which is why MDT-BRIDGE choosing resection rate as its primary endpoint matters more than any subgroup pCR figure (Reck et al., 2024).

### 5.3 Toxicity acts on operability through reserve, not through anatomy

Treatment-related cancellation of surgery of 1%–2% is genuinely reassuring, and it is reassuring within a denominator of fit patients (Yue et al., 2025a; Mitsudomi et al., 2025). The borderline patient differs in margin rather than in kind. A grade 3 colitis with 4 weeks of corticosteroid, a grade 2 pneumonitis that resolves slowly, or a 4-week delay for logistical reasons compounded by deconditioning, all subtract from a physiological reserve that was already close to the operative threshold (Brunelli et al., 2009). Surgical feasibility was preserved across the three reported NeoCOAST-2 arms, at 93.0%–94.4%, but those arms enrolled 54 to 74 patients each without a borderline resectable subgroup and without a randomised comparator (Cascone et al., 2025). None of the six pivotal trials reported a functional outcome: no postoperative pulmonary function, no exercise capacity, no patient-reported physical function stratified by whether an immune-mediated event occurred. That omission, not the cancellation rate, is the gap that matters for this population.

### 5.4 Anchoring the acceptable risk

ASCO guidance requires that R0 resection be judged achievable, that N3 disease be excluded by multidisciplinary consensus, and that perioperative 90-day mortality not be expected to exceed approximately 5% for surgery to form part of stage III multimodality treatment (Daly et al., 2022). Observed 90-day mortality of 4.0% in the pembrolizumab arm of KEYNOTE-671 sits inside that limit while leaving little headroom (Wakelee et al., 2023). That guidance predates routine perioperative immunotherapy, so it was written without the corticosteroid exposure, the delayed operations and the interstitial lung disease events that now form part of the induction pathway. Applying an unmodified 5% threshold to a population selected for contested resectability, after four cycles of chemoimmunotherapy, extrapolates further than the guideline was designed to support.

### 5.5 Prognostic is not predictive

PD-L1 expression, baseline circulating tumour DNA and on-treatment clearance correlate with event-free and overall survival after neoadjuvant therapy, and pathological response is associated with survival across pooled neoadjuvant datasets, which is what supports its use as a surrogate (Forde et al., 2025; Deutsch et al., 2024). All of that is prognostic or surrogate information. None of it establishes that a biomarker predicts differential benefit from surgery against definitive chemoradiation, because that claim requires a treatment-by-biomarker interaction test in a randomised comparison of the two strategies, and no such comparison has been performed in a borderline resectable population. The same caution applies to subgroup observations, including the numerically larger event-free survival effect seen in squamous histology in the KEYNOTE-671 subgroup analysis (Spicer et al., 2024). For the emerging agents the position is weaker still: TROP2, CD73, Siglec-15 and B7-H3 expression are all hypothesis-generating rather than validated predictive markers (Cascone et al., 2025; Aggarwal et al., 2022; Wang et al., 2019).

### 5.6 Why stage IIB is discussed alongside stage III

This review draws safety data from trials that enrolled from stage II onwards, because that is how the pivotal trials were designed: KEYNOTE-671 and RATIONALE-315 enrolled from stage II, AEGEAN from stage IIA and CheckMate 77T from stage IIA (Wakelee et al., 2023; Heymach et al., 2023; Cascone et al., 2024; Yue et al., 2025a). MDT-BRIDGE likewise enrols from stage IIB (Reck et al., 2024). Restricting the safety discussion to stage III alone would discard most of the reported toxicity denominators while changing none of the conclusions, since immune-mediated events are not stage-specific. Where an efficacy claim depends on stage, we have stated the enrolled population.

## 6 Limitations of the current evidence base

This is a narrative review. Searches of PubMed, ClinicalTrials.gov, ASCO and WCLC proceedings and company announcements were conducted to August 2026, without a PRISMA-compliant search strategy, without duplicate independent screening and without formal risk-of-bias assessment. Selection of the emerging agents discussed in Section 4 reflects our judgement of surgical relevance rather than a prespecified protocol.

The central limitation is definitional. The borderline resectable definition adopted here, drawn from the EORTC consensus and survey (Dingemans et al., 2026; Houda et al., 2025), has not been retrofitted to any pivotal trial dataset. Every safety and feasibility figure we quote therefore describes a fitter, more unambiguously resectable population than the one the review is about, and the direction of that bias is predictable: toxicity consequences will be underestimated and surgical feasibility overestimated relative to a genuinely borderline cohort.

Evidence levels are heterogeneous and we have flagged them individually rather than averaging over them. The RATIONALE-315 surgical values in Table 3 are congress-reported (Yue et al., 2025b), and four emerging-agent programmes rest on conference abstracts or company statements (Gettinger et al., 2023; Spigel et al., 2024; BioNTech and Myers Squibb, 2026; Hiltermann et al., 2024), including the negative COSTAR Lung result, which we accept on company-reported evidence while noting that we would not accept a positive result on the same basis. Several values are genuinely absent: aggregate immune-mediated event rates in CheckMate 816 and CheckMate 77T, arm-level grade 3 or higher immune-mediated events in NeoCOAST-2, and cancellation data in NEOTORCH and CheckMate 77T. We have recorded these as not reported rather than substituting adjacent figures.

Cross-trial safety comparison is limited by non-harmonised terminology, adjudication and reporting windows, and the AEGEAN grade 3 or higher column derives from an all-cause rather than a treatment-related table (Heymach et al., 2023). Two cited articles carry formal corrections, affecting an author name in the NEOTORCH publication (Lu et al., 2024) and an agent label in the adenosine pathway review (Allard et al., 2020). No cited article is retracted and no expression of concern was identified as of 25 August 2026.

## 7 Conclusion

Preoperative PD-1 and PD-L1 blockade has a reproducible pathological and event-free survival benefit across six phase 3 trials, with pCR of 17.2%–40.7%, event-free survival hazard ratios of 0.40–0.68, and treatment-related cancellation of surgery below 2% wherever it was reported (Forde et al., 2022; Wakelee et al., 2023; Heymach et al., 2023; Cascone et al., 2024; Lu et al., 2024; Yue et al., 2025a; Mitsudomi et al., 2025). Aggregate toxicity is therefore not the constraint on using these regimens in borderline resectable disease. The constraint is the interaction between a grade 3 immune event, a corticosteroid course, a delay of several weeks and a functional reserve that was marginal before treatment started, and no pivotal trial measured that interaction.

The emerging landscape has narrowed rather than widened over the period covered by this revision. Monospecific TIGIT blockade failed in three randomised settings including unresectable stage III disease (Peters et al., 2026; Rudin et al., 2024; Wakelee et al., 2026), and the most advanced TIM-3 programme was discontinued after a negative phase 3 read-out reported by its sponsor (ClinicalTrials.gov, 2026b; GSK plc, 2025). What remains credible for preoperative testing is a shorter list than 3 years ago. The NKG2A, CD73 and TROP2 combinations from the NeoCOAST platform have neoadjuvant pathological response signals with preserved surgical feasibility in cohorts of 54–74 patients (Cascone et al., 2023; Cascone et al., 2025). PD-1 with VEGF bispecific blockade has a positive phase 3 result in advanced disease and no surgical data (Xiong et al., 2025).

Mechanistically, the reason toxicity matters more in this population than in the trial populations is that immune-mediated events act on functional operability rather than on technical resectability. A pneumonitis does not change a T or N descriptor; it changes whether the patient tolerates the resection that the descriptor permits (Brunelli et al., 2009). That is why cancellation rates measured in fit cohorts do not transfer, and why the useful endpoints for the next-generation of trials are resection rate after multidisciplinary reassessment, time from diagnosis to definitive treatment, and postoperative functional recovery.

What is needed is not another single-arm neoadjuvant platform but prospective cohorts in which borderline status is defined by a multidisciplinary team before treatment and in which functional outcomes are reported alongside pathological ones. MDT-BRIDGE, with resection rate as its primary endpoint, and NEOINSPIRE, with the first randomised preoperative comparison of a bispecific against a PD-1 antibody, are the first trials constructed that way, and neither has reported (Reck et al., 2024; ClinicalTrials.gov, 2026a). Until they do, the decision to give a borderline resectable patient preoperative checkpoint inhibition rests on toxicity data generated in patients who were never borderline.
